# Impacto de la pandemia de COVID-19 sobre la utilización de la medición de la HbA_1c_ y sus resultados en pacientes ambulatorios adultos y pediátricos con diabetes

**DOI:** 10.1515/almed-2023-0012

**Published:** 2023-03-06

**Authors:** Paloma Oliver, Marina Pellicer, Daniel Prieto, Jorge Diaz-Garzon, Roberto Mora, Ileana Tomoiu, Noemi Gonzalez, Atilano Carcavilla, Isabel Gonzalez-Casado, Itsaso Losantos, Antonio Buño, Pilar Fernandez-Calle

**Affiliations:** Servicio de Análisis Clínicos, Hospital Universitario La Paz, Madrid, España; Servicio de Endocrinología, Hospital Universitario La Paz, Madrid, España; Servicio de Endocrinología Pediátrica, Hospital Universitario La Paz, Madrid, España; Departamento de Bioestadística, Hospital Universitario La Paz, Madrid, España

**Keywords:** COVID-19, diabetes mellitus, técnicas y procedimientos diagnósticos, telemedicina

## Abstract

**Objetivos:**

La diabetes mellitus incrementa los riesgos y complicaciones asociadas a la COVID-19. Una de las principales consecuencias de la pandemia ha sido la drástica reducción de las consultas presenciales. El objetivo de este estudio es evaluar el impacto que ha tenido la pandemia de COVID-19 en la gestión de la determinación de HbA_1c_ y sus resultados en pacientes ambulatorios adultos y pediátricos con diabetes, teniendo en cuenta tanto la medición realizada en el laboratorio como las pruebas de laboratorio en el lugar de asistencia o *point-of-care testing* (POCT).

**Métodos:**

Se realizó un estudio observacional retrospectivo que incluyó pacientes de las Unidades de Diabetes Pediátrica y de Adultos. A través del sistema de información del laboratorio, se extrajeron los resultados de HbA_1c_ obtenidos en el laboratorio y los resultados de POCT en un periodo de tres años (2019–2021).

**Resultados:**

El número de mediciones de HbA_1c_ se redujo considerablemente tras el confinamiento. En poco tiempo, los pacientes pediátricos volvieron a recibir su asistencia médica habitual. El número de mediciones de HbA_1c_ fue aumentando paulatinamente en los adultos, especialmente POCT. En general, los valores de HbA_1c_ fueron inferiores en los pacientes pediátricos que en los adultos (p<0,001). Los valores de HbA_1c_ en niños (p<0,001) y adultos (p=0,002) se redujeron tras la pandemia con respecto al periodo previo a la misma, aunque fueron inferiores al valor de referencia del cambio de la HbA_1c_. El porcentaje de resultados de HbA_1c_ superiores al 8% se mantuvo estable durante el periodo de estudio.

**Conclusiones:**

Los sistemas de monitorización continua de la glucosa y la telemedicina fueron cruciales, habiéndose producido incluso una mejoría respecto a los niveles de HbA_1c_. Durante el confinamiento, a los pacientes con mejor control metabólico, las pruebas analíticas se les realizaron en el laboratorio, mientras que a los pacientes con peor control o una situación clínica grave se les realizaron mediante POCT en las Unidades de Diabetes. En los pacientes adultos, el retorno a la asistencia habitual previa a la pandemia se produjo de forma lenta y gradual, ya que presentaban mayor riesgo de morbimortalidad asociado a la COVID-19. La coordinación entre todos los profesionales sanitarios fue esencial a la hora de garantizar la mejor atención posible, especialmente en escenarios complejos, como la pandemia de COVID-19.

## Introducción

La diabetes mellitus (DM) es una de las principales causas de morbimortalidad en todo el mundo. Esta enfermedad se asocia a numerosas complicaciones, que influyen en la gravedad de la enfermedad. Existen estudios que muestran que una elevada concentración de glucosa en sangre y la DM desempeñan un papel relevante en la mortalidad y la gravedad de las infecciones víricas, como las producidas por el coronavirus causante del síndrome respiratorio de Oriente Medio (MERS-CoV), el H1N1 (gripe A) y el coronavirus causante del síndrome respiratorio agudo severo (SARS-CoV). Tanto la DM tipo 1 como la tipo 2 incrementan los riesgos y las complicaciones asociadas a la COVID-19, siendo el riesgo todavía más elevado en los pacientes de mayor edad [1].

Una de las consecuencias de la pandemia de COVID-19 en el sistema sanitario ha sido la drástica reducción de las consultas presenciales, lo que ha suscitado inquietud sobre el impacto que este hecho podría tener en el manejo y control de los pacientes crónicos. Se ha observado una reducción del control metabólico, especialmente durante el confinamiento. En este contexto, la asistencia se redirigió hacia la telemedicina para compensar la reducción de las consultas presenciales [2]. La disminución de las consultas habituales puede dar lugar a un peor control metabólico y a complicaciones que no son tratadas a su debido tiempo [3]. Para evitar dichas complicaciones, es importante que las personas con diabetes midan regularmente sus concentraciones de glucosa en sangre, asistan a sus citas médicas, tengan acceso al tratamiento médico adecuado, sean físicamente activas y lleven una dieta saludable [4].

Asimismo, la atención secundaria que los pacientes con DM y COVID-19 requieren tiene un mayor coste que la que precisan los pacientes con COVID-19 y sin DM [3]. Algunos autores también han hecho hincapié en la importancia de reforzar la prevención y el tratamiento adecuado de la DM, con especial énfasis en la prevención de la COVID-19 en pacientes con DM [3].

El Hospital Universitario La Paz es un hospital público terciario ubicado en Madrid, cuyas especialidades cuentan con un amplio reconocimiento. Se trata de uno de los hospitales más grandes de España, dotado de unas 1.300 camas y con varios centros satélites que ofrecen diferentes especialidades y 23 centros de atención primaria. El Servicio de Análisis Clínicos de este hospital consta de una Unidad de Pruebas de Laboratorio en el Lugar de Asistencia (pruebas de cabecera o *Point-of-Care Testing*, POCT), que ha coordinado la red de POCT del hospital durante los últimos 23 años. Esta red de POCT cuenta con dos analizadores en las Unidades de Diabetes Pediátrica y de Adultos [5]. El seguimiento de los pacientes con diabetes consiste en la determinación de HbA_1c_, en el laboratorio oa través de POCT en nuestro hospital. De este modo, realizar una evaluación integral del manejo de la determinación de HbA_1c_ y de los resultados obtenidos en las dos localizaciones es una herramienta eficaz a la hora de evaluar la práctica clínica, siendo también necesaria para obtener una visión general del manejo de la diabetes.

Desde el confinamiento, que se inició el 11 de marzo de 2020 en Madrid, las consultas hospitalarias habituales de los pacientes con diabetes se han reducido drásticamente, lo que se ha traducido en una asistencia más centrada en la telemedicina a causa de la pandemia de COVID-19.

El objetivo de este estudio es evaluar el impacto que ha tenido la pandemia de COVID-19 en la gestión de la determinación de HbA_1c_ y sus resultados en los pacientes ambulatorios adultos y pediátricos con diabetes, teniendo en cuenta los resultados obtenidos en el laboratorio y los resultados de POCT.

## Materiales y métodos

Este trabajo es un estudio observacional retrospectivo que incluyó pacientes de las Unidades de Diabetes Pediátrica y de Adultos.

Los resultados de HbA_1c_ obtenidos en el laboratorio (Tosoh G11; Horiba Medical) y los resultados de POCT (DCA Vantage; Siemens Healthineers) entre 2019 y 2021 fueron extraídos anónimamente utilizando el el sistema de información del laboratorio (LabTrak; Intersystems).

Todas estas determinaciones están acreditadas por la *International Organization for Standardization* (ISO) 15189 y la ISO 22870 de la Entidad Nacional de Acreditación en España (ENAC). Se realizó un estudio de comparación de los resultados de HbA_1c_ obtenidos en el laboratorio central y los resultados de POCT del DCA Vantage, confirmándose su intercambiabilidad. Asimismo, se realizó una evaluación de los procedimientos de aseguramiento de la calidad interna y externa realizados tanto en el laboratorio como en POCT, con el objeto de comprobar si cumplían las especificaciones de calidad establecidas por el laboratorio.

Del mismo modo, el laboratorio estableció una serie de indicadores de calidad mensuales para realizar un seguimiento y evaluar el rendimiento de los aspectos críticos de los procesos analíticos y extra-analíticos.

A continuación, se enumeran las variables seleccionadas relacionadas con la gestión de la determinación de HbA_1c_ y sus resultados:

### Medición de la HbA_1c_

– Número total de determinaciones de HbA_1c_ realizadas (n).
– Razón del número de mediciones de HbA_1c_ en el laboratorio/número de determinaciones de HbA_1c_ en POCT (%).
– Número total de pacientes atendidos en las consultas (n).
– Razón de mediciones de HbA_1c_/paciente (n).

### Resultados de HbA_1c_

– Resultados de HbA_1c_, expresados como valores medios y desviación estándar (DE).
– Resultados de HbA_1c_ >8% (expresados como porcentaje de resultados de HbA_1c_ >8% con respecto a la totalidad de resultados de HbA_1c_).

### Métodos estadísticos

En el presente estudio, se tuvieron en cuenta dos periodos de tiempo: el periodo pre-pandemia, entre enero de 2019 y febrero de 2020, y el periodo post-pandemia, entre marzo de 2020 y diciembre de 2021. La normalidad en la distribución de los resultados de HbA_1c_ se evaluó mediante la prueba de Kolmogorov–Smirnov. Se realizó un análisis descriptivo de los resultados de HbA_1c_, calculando los resultados medios y DE. Las diferencias en los resultados de HbA_1c_ entre los pacientes pediátricos y adultos, así como entre las pruebas de laboratorio y POCT, se analizaron con la prueba *t* de Student, teniendo también en cuenta el valor de referencia del cambio (VRC) de la HbA_1c_. El VRC se calculó aplicando la fórmula VRC = 2^1/2^ × Z × (CVI^2^+CVA^2^)^1/2^, con una variabilidad biológica intraindividual (CVI) de HbA_1c_ del 1,6%, y un coeficiente de variabilidad analítica anual (CVA) del 2,8% para una probabilidad del 95% [6]. Se aplicaron modelos lineales generalizados para evaluar el efecto de la pandemia en los pacientes pediátricos y adultos. Un valor *p* inferior a 0,05 fue considerado estadísticamente significativo.

## Resultados

### Utilización de la medición de la HbA_1c_

**Figura 1:**
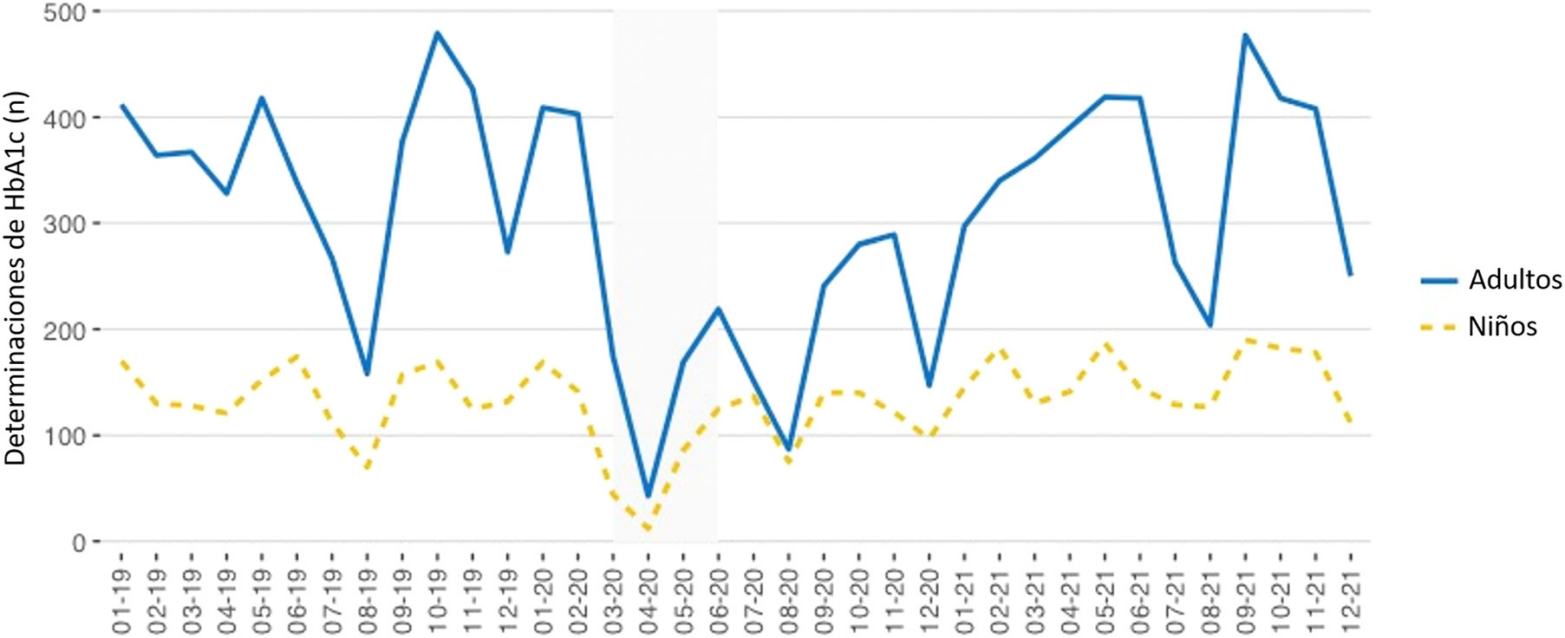
Eje Y: Número total de determinaciones de HbA_1c_ (laboratorio + POCT) realizadas en cada Unidad de Diabetes. Eje X: Tiempo (mes-año). El periodo de estricto confinamiento en Madrid corresponde al área sombreada (marzo-mayo 2020).

**Figura 2:**
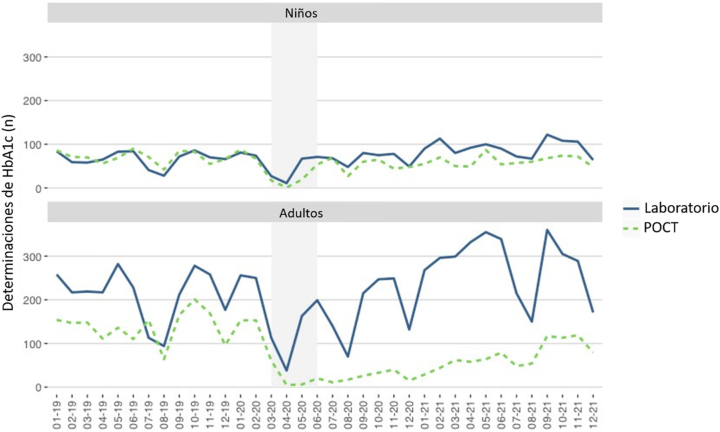
Eje Y: Número de determinaciones de HbA_1c_ de pacientes pediátricos y adultos en el laboratorio y POCT. Eje X: Tiempo (mes-año). El periodo de estricto confinamiento en Madrid corresponde al área sombreada (marzo-mayo 2020).

Las Figuras 1–2 y la Figura Suplementaria 1 muestran el número de determinaciones de HbA_1c_ (laboratorio y POCT) realizadas en las Unidades de Diabetes Pediátrica y de Adultos.

En la Unidad Pediátrica, se realizó un total de 4.776 determinaciones de HbA_1c_, mientras que en la Unidad de Adultos se llevaron a cabo 11.065 determinaciones. Durante el periodo de estudio, 1.042 niños y 1.767 adultos fueron atendidos, con 1,5 mediciones anuales por paciente pediátrico, y 2,1 mediciones por paciente adulto.

Durante el confinamiento estricto (marzo+abril+mayo), en los pacientes pediátricos, se realizó un total de 81 determinaciones en el laboratorio (3+11+67) y 21 en POCT (1+1+19). Con respecto a los adultos, se llevaron a cabo 221 determinaciones en el laboratorio (20+38+163) y 11 en POCT (0+5+6).

Previamente al mes de marzo de 2020, el número de mediciones de HbA_1c_ era estable en los dos contextos clínicos. Tras el 11 de marzo de 2020, el número de mediciones de HbA_1c_ disminuyó notablemente. En las Figuras 1–2 y en la Figura Suplementaria 1 se puede observar que, en junio de 2020, los pacientes pediátricos volvieron a recibir la atención médica habitual. Sin embargo, el número de determinaciones de HbA_1c_ se fue incrementando paulatinamente en los adultos, especialmente en POCT.

### Resultados de HbA_1c_

**Figura 3:**
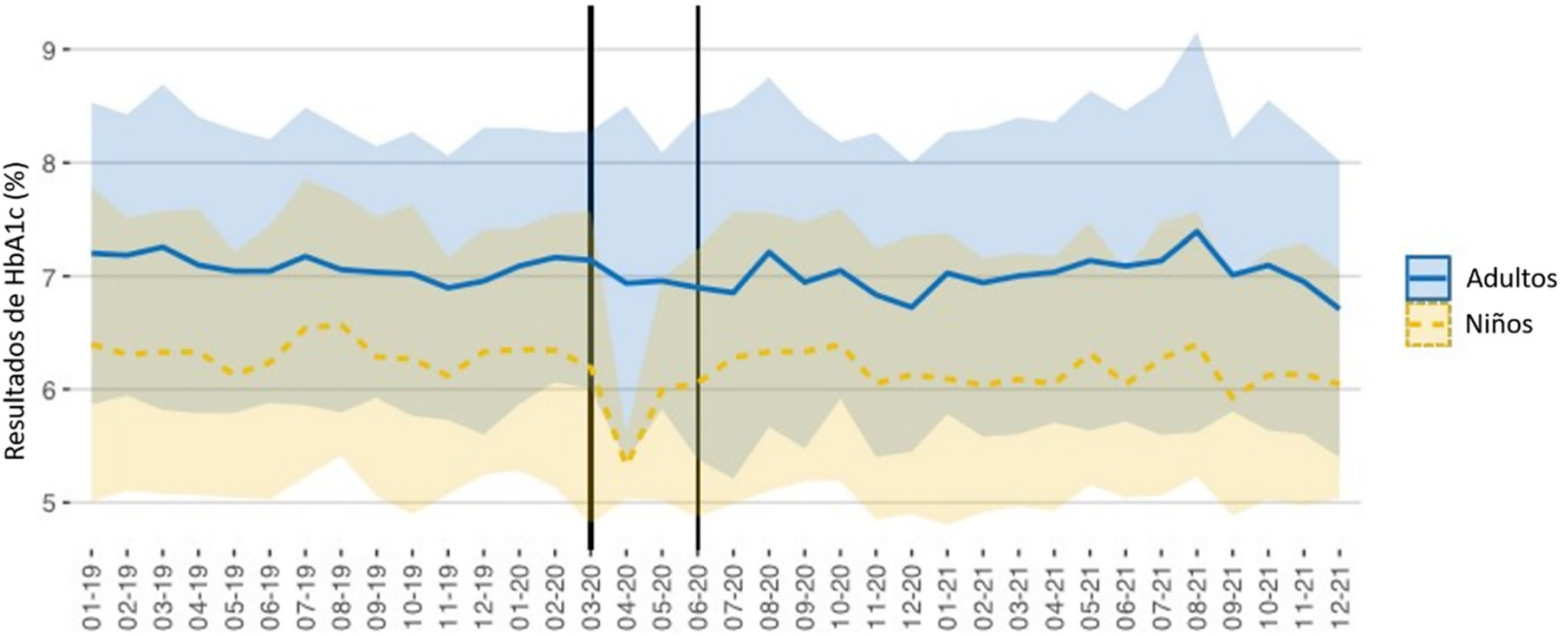
Eje Y: Resultados de HbA_1c_ (media ± 1DS) obtenidos en pacientes pediátricos y adultos (laboratorio + POCT). Eje X: Tiempo (mes-año). El periodo de estricto confinamiento en Madrid corresponde al área sombreada (marzo-mayo 2020).

**Figura 4:**
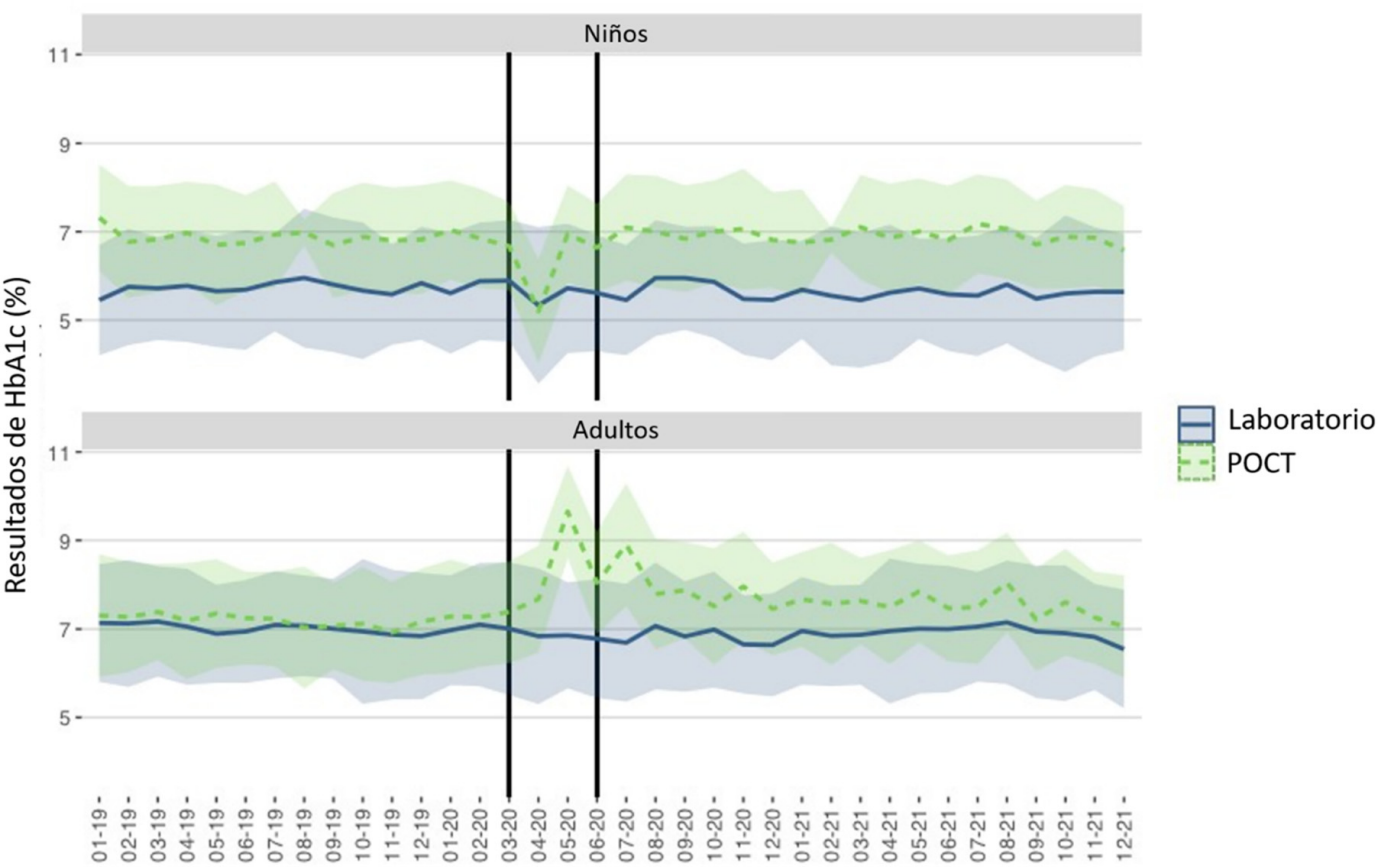
Eje Y: Resultados de HbA_1c_ (media ± 1DS) obtenidos en pacientes pediátricos y adultos en el laboratorio y POCT. Eje X: Tiempo (mes-año). El periodo de estricto confinamiento en Madrid corresponde al área sombreada (marzo-mayo 2020).

Las Figuras 3–4 muestran el valor medio y DE de todos los resultados de HbA_1c_ (laboratorio y POCT) solicitados en la Unidad de Diabetes Pediátrica y de Adultos durante el periodo de estudio.

En general, los resultados de HbA_1c_ fueron inferiores en los pacientes pediátricos (6,21%; DE: 1,18), frente a los adultos (7,05%; DE: 1,32). La diferencia fue significativa, según la prueba *t* de Student (p<0,001), tomando 8,9% como el VRC de la HbA_1c_.

Teniendo en cuenta los periodos pre-pandemia y post-pandemia, observamos una diferencia significativa en los pacientes pediátricos con respecto a los resultados de HbA_1c_: 6,31% (DE: 1,21) frente a 6,15% (DS: 1,15), p<0,001; así como en los pacientes adultos: 7,09% (DE: 1,25) frente a 7,01% (DE: 1,39), p=0,002. No obstante, las diferencias fueron inferiores al VRC.

Tomando febrero de 2020 como el mes de referencia del periodo pre-pandemia, en los pacientes pediátricos podemos observar que se produjo una reducción significativa en el valor medio de HbA_1c_ en abril (p=0,003), mayo (p=0,026) y junio (p=0,043). A partir de julio de 2020, los resultados fueron similares a los obtenidos en el periodo prepandemia. Con respecto a los pacientes adultos, observamos una tendencia similar en junio (p=0,020) y julio (p=0,017), volviendo a observar resultados similares a los del periodo pre-pandemia a partir de agosto de 2020.

Al comparar los resultados de HbA_1c_ obtenidos en el laboratorio frente a los resultados de POCT en pacientes pediátricos, observamos diferencias significativas en los resultados obtenidos en el laboratorio (p=0,001), pero no en los obtenidos en POCT (p=0,176), a lo largo del periodo de estudio. Tomando febrero de 2020 como referencia, no se produjeron diferencias en los meses siguientes, ni en los valores obtenidos en el laboratorio, ni en los resultados de POCT.

En adultos, hubo diferencias significativas en los resultados de HbA_1c_ obtenidos en el laboratorio (p=0,001) frente a los resultados de POCT (p=0,009) a lo largo del periodo de estudio. Tomando febrero de 2020 como referencia, se pueden observar diferencias en los valores de laboratorio en mayo (p=0,041), junio (p=0,005) y julio (p=0,001). También hubo diferencias en los resultados de POCT en mayo (p=0,002) y julio (p=0,004). A partir de agosto de 2020, los resultados fueron similares a los obtenidos en el periodo pre-pandemia.

**Figura 5:**
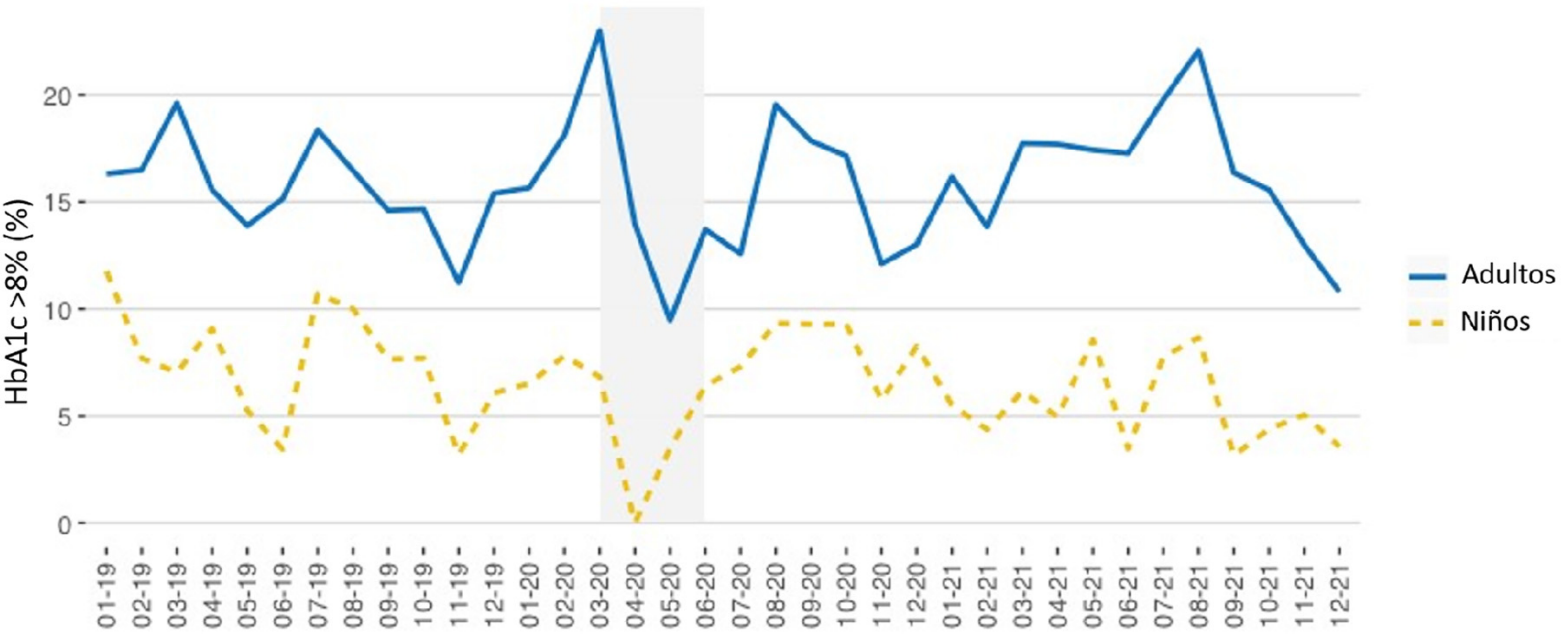
Eje Y: Porcentajes de HbA_1c_ >8% (laboratorio y POCT) obtenidos en pacientes pediátricos y adultos. Eje X: Tiempo (mes-año). El periodo de estricto confinamiento en Madrid corresponde al área sombreada (marzo-mayo 2020).

**Figura 6:**
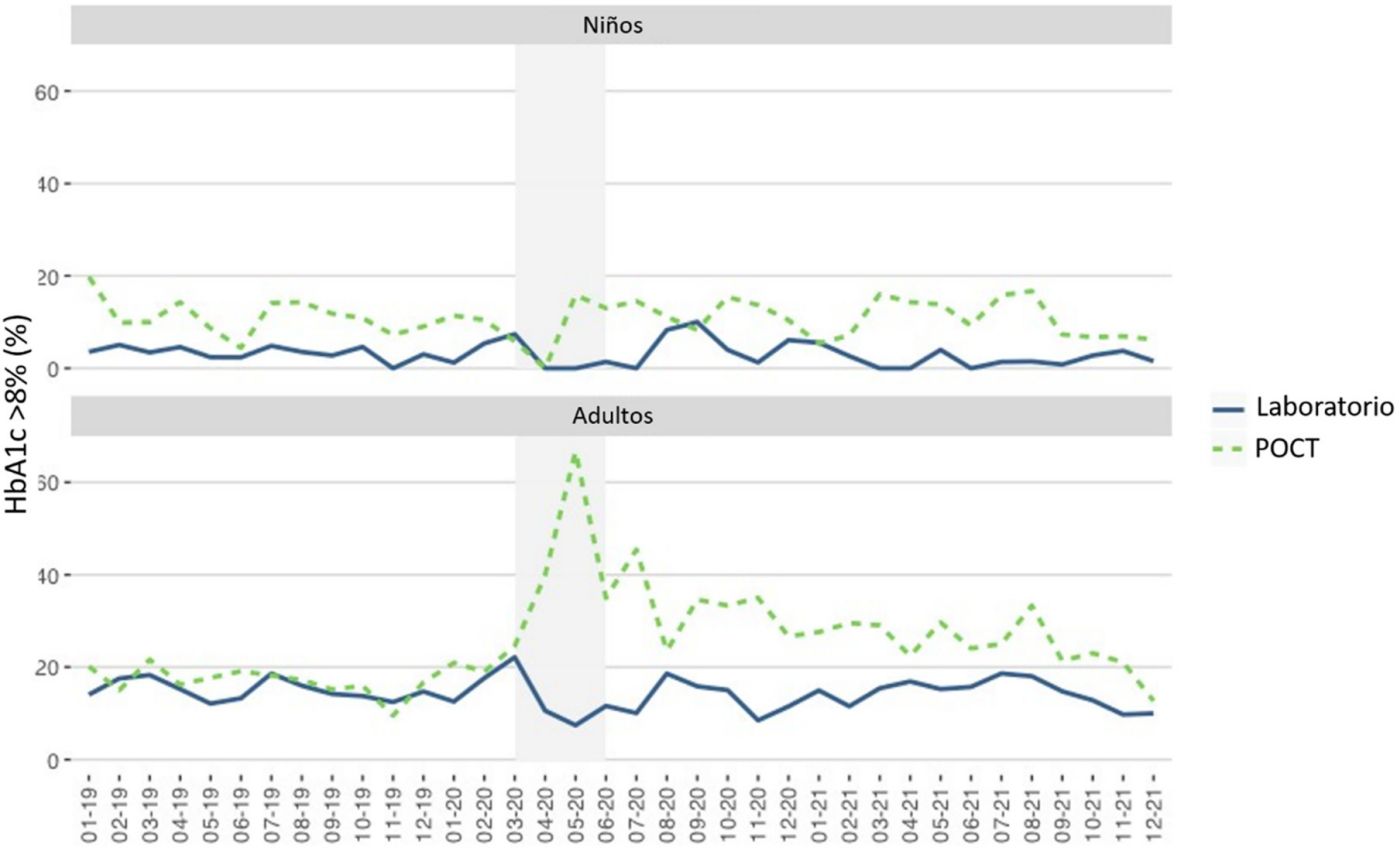
Eje Y: Porcentajes de HbA_1c_ >8% obtenidos en pacientes pediátricos y adultos en el laboratorio y POCT. Eje X: Tiempo (mes-año). El periodo de estricto confinamiento en Madrid corresponde al área sombreada (marzo-mayo 2020).

Las Figuras 5–6 muestran los resultados de HbA_1c_ superiores a 8% (63,9 mmol/mol IFCC) obtenidos en los pacientes pediátricos y adultos.

En general, el porcentaje de resultados de HbA_1c_ por encima de 8% permaneció estable durante el periodo de estudio. Durante el confinamiento estricto, los resultados disminuyeron en las dos poblaciones y no se hallaron pacientes pediátricos con resultados de HbA_1c_ superiores a 8% hasta mayo de 2020, cuando se identificaron cuatro pacientes con valores de HbA_1c_ >8% en las determinaciones de POCT realizadas en la consulta de la Unidad de Diabetes Pediátrica.

En los pacientes adultos, el porcentaje de valores de HbA_1c_ >8% permaneció estable en el laboratorio, incluso durante el confinamiento estricto. Sin embargo, en los resultados de POCT, observamos un incremento del 40% en abril, y del 67% en mayo de 2020.

## Discusión

Este estudio evalúa las determinaciones de HbA_1c_ realizadas tanto en el laboratorio como en POCT, para obtener una perspectiva global del manejo de los pacientes pediátricos y adultos con diabetes. La mayoría de las publicaciones se centran en las determinaciones llevadas a cabo en el laboratorio, excluyendo los resultados de POCT [5]. El desarrollo de una red consolidada y acreditada de POCT en nuestro hospital a lo largo de los últimos 23 años nos ha permitido realizar una evaluación de todo el proceso, detectando desviaciones que podrían tener un impacto en la atención al paciente.

Durante el confinamiento (marzo-mayo de 2020), el seguimiento de los pacientes con diabetes se realizó a través de la telemedicina, sin que se produjeran pérdidas de seguimiento. Ello explica el descenso significativo en el número total de determinaciones de HbA_1c_ observado durante dicho periodo. Las consultas hospitalarias presenciales se fueron retomando a partir de junio de 2020. La vuelta a la situación previa a la pandemia se produjo a menor velocidad en los adultos, ya que se observó una mayor incidencia de mortalidad y del número de pacientes críticos entre las personas de mayor edad o con comorbilidades como la DM, la hipertensión o el cáncer [1]. Estos pacientes preferían acudir a la sala de extracciones del hospital para que se enviaran sus muestras al laboratorio y posteriormente asistir a una teleconsulta.

En relación a los resultados de HbA_1c_, estos no aumentaron en el periodo post-pandemia. Aproximadamente, el 45% de los pacientes adultos y el 90% de los pacientes pediátricos con DM tipo 1 se sometieron a una monitorización continua de glucosa desde 2020. A pesar de las dificultades de la pandemia de COVID-19, la monitorización a distancia y el elevado nivel de auto-seguimiento de la diabetes contribuyeron a mejorar el control metabólico. Estos resultados coinciden con los obtenidos en otros estudios [7]. Luzi *y col.* [8] detectaron una reducción de HbA_1c_ durante el confinamiento en los pacientes que utilizaron sistemas de monitorización continua de glucosa como método de gestión de la diabetes durante dicho periodo. Alharthi *y col.* [9] observaron que aquellos que asistieron a teleconsultas durante el confinamiento mejoraron sus parámetros glucémicos. Por otro lado, no se produjeron cambios significativos en aquellos pacientes que no asistieron a teleconsultas. Estos resultados demuestran la efectividad clínica de la telemedicina en el manejo de la diabetes.

Aunque no haya evidencia de la asociación negativa entre el descenso de las consultas externas y el control glucémico en 2020, aún existe inquietud sobre los posibles efectos que ello haya podido tener sobre la detección, el seguimiento y el control glucémico, dado que un gran número de las consultas presenciales se pospusieron durante la pandemia, y no todos los pacientes tuvieron el mismo acceso a la telemedicina. Es necesario retomar las consultas presenciales para todos los pacientes con patologías crónicas, con el fin de garantizar su correcto seguimiento y control y prevenir consecuencias negativas en el futuro [2].

Por otro lado, otros estudios han reportado una pérdida de control glucémico durante el confinamiento, con las consecuencias derivadas de ello [1]. Con respecto a la situación inicial, los autores detectaron un incremento del 2,26% en los resultados de HbA_1c_, tras 30 días de confinamiento, y un aumento del 3,68% tras 45 días de confinamiento. La tasa de complicaciones aumentó un 2,9% en las neuropatías periféricas, 2,8% en la retinopatía diabética, 0,5% en el ictus, 0,9% en los infartos, y un incremento del 14,2% y del 9,3% en la proteinuria y la microalbuminuria, respectivamente. Este cambio representó un aumento del número de pacientes con DM y complicaciones derivadas de dicha patología [1]. Pardhan *y col.* [4] observaron que el autoaislamiento influyó en casi todos los factores que afectan al autocontrol de la diabetes. Para mejorar el acceso a los fármacos para la diabetes y promover una dieta saludable entre los pacientes que precisan autoaislamiento, es esencial garantizar que dichos pacientes tengan capacidad para control su diabetes de manera efectiva.

Con respecto a los pacientes pediátricos, Predieri *y col.* [10] evaluaron a pacientes con DM tipo 1 que hicieron uso de sistemas de monitorización continua de glucosa con anterioridad al cierre de los centros escolares y posteriormente al confinamiento. El control glucémico mejoró durante el confinamiento. A pesar de que los pacientes se encontraban confinados en sus casas y realizaban ejercicio físico limitado, el uso de los sistemas de monitorización continua de la glucosa, la vigilancia parental continua y la telemedicina mostraron efectos beneficiosos en el cuidado de los pacientes. Hakonen *y col.* [11] obtuvieron resultados similares. El control glucémico en los niños con DM tipo 1 no empeoró durante el confinamiento, y los pacientes que utilizaban bombas de insulina incluso mejoraron su control, lo que sugiere que la limitación del contacto social podría haber permitido a las familias utilizar la bomba de insulina con mayor precisión, dado que los niños no realizaban actividades fuera de casa.

Finalmente, el incremento de los valores de HbA_1c_ por encima del 8% observado en nuestro estudio en los resultados de POCT en adultos en abril y mayo de 2020 estuvo relacionado con aquellos pacientes más graves que siguieron recibiendo asistencia a través del Servicio de Urgencia: debut de diabetes, cetoacidosis o crisis hiperosmolar. Dichos pacientes fueron derivados directamente a la Unidad de Diabetes para una consulta presencial.

## Conclusiones

En resumen, el manejo de los pacientes con diabetes durante la pandemia de COVID-19 ha supuesto todo un reto. En nuestro hospital, los sistemas de monitorización continua de glucosa y la telemedicina han sido cruciales, habiendo incluso mejorado los resultados de HbA_1c_. Durante el confinamiento, a los pacientes con mejor control metabólico se les manejó desde el laboratorio. Por otro lado, a los pacientes con peor control o una situación clínica grave se les realizaron pruebas de POCT en las Unidades de Diabetes. Los adultos volvieron paulatinamente a una situación similar a la previa a la pandemia, ya que tenían mayor riesgo de morbimortalidad asociada a la COVID-19 y se habían tomado más precauciones.

La coordinación entre todos los profesionales sanitarios ha sido un pilar fundamental a la hora de ofrecer la mejor gestión posible, centrada en el estado clínico de los pacientes, especialmente en situaciones complejas como la pandemia de COVID-19. La combinación adecuada de los métodos de laboratorio y el POCT para la monitorización de la HbA_1c_ junto a las nuevas estrategias de manejo, como la telemedicina, contribuyó a alcanzar estos resultados.

## Supplementary Material

Supplementary Material

## Material Suplementario

La versión en línea de este artículo ofrece material suplementario (https://doi.org/10.1515/almed-2023-0012).
