# Supplementary material for: Impacto de la pandemia de COVID-19 sobre la utilización de la medición de la HbA1c y sus resultados en pacientes ambulatorios adultos y pediátricos con diabetes
Source: Adv Lab Med. 2023 Mar 6;4(1):112–9. [Article in Spanish] doi: 10.1515/almed-2023-0012 (PMC10197185; doi:10.1515/almed-2023-0012)
Supplement: Supplementary file 1 — Supplementary Material [file j_almed-2023-0012_suppl.docx]

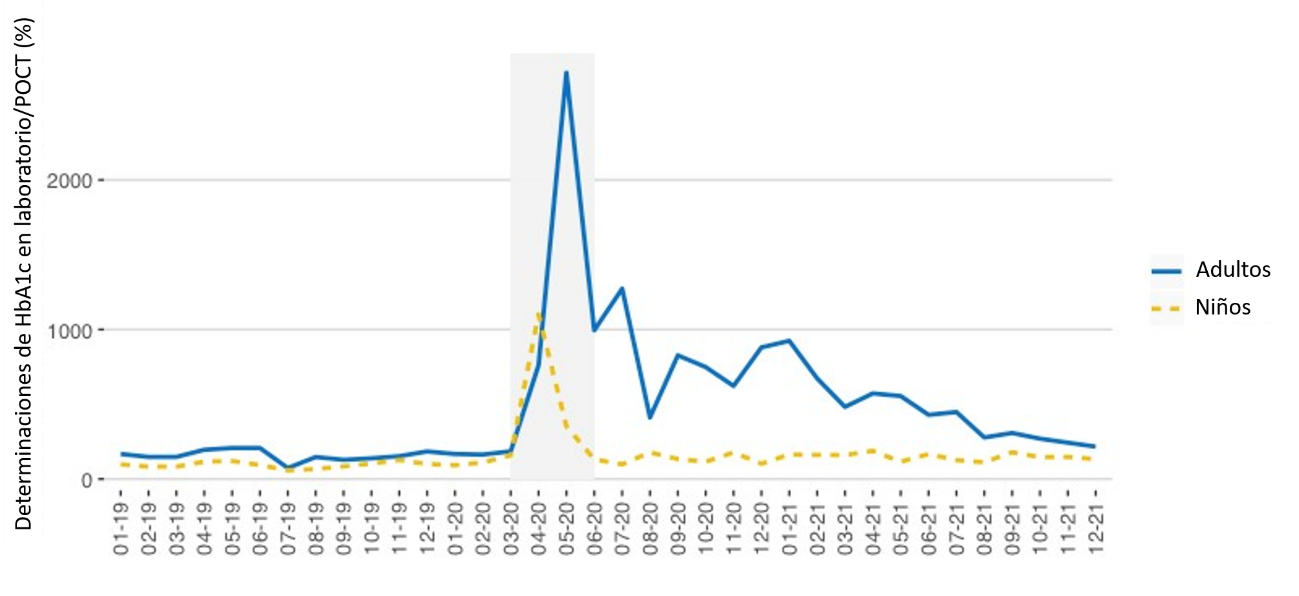


**Figura suplementaria 1.** Eje Y: Razón de determinaciones de HbA1c realizadas en el laboratorio / POCT (%) en cada Unidad de Diabetes. Eje X: Tiempo (mes-año). El periodo de estricto confinamiento en Madrid corresponde al área sombreada (marzo-mayo 2020).
